# Human herpesvirus type 2 infection of primary murine astrocytes causes disruption of the mitochondrial network and remodeling of the actin cytoskeleton: an *in vitro* morphological study

**DOI:** 10.1007/s00705-021-05025-x

**Published:** 2021-03-14

**Authors:** Anna Słońska, Joanna Cymerys, Marcin Chodkowski, Piotr Bąska, Małgorzata Krzyżowska, Marcin W. Bańbura

**Affiliations:** 1 Division of Microbiology, Department of Preclinical Sciences, Institute of Veterinary Medicine, Warsaw University of Life Sciences, Ciszewskiego 8, 02-786 Warsaw, Poland; 2 Military Institute of Hygiene and Epidemiology, Kozielska 4, 01-163 Warsaw, Poland

## Abstract

Herpesviruses are capable of infecting not only neurons, where they establish latent infection, but also astrocytes. Since astrocytes are important for the functioning of the central nervous system (CNS), their infection may lead to serious neurological disorders. Thus, in the present study we investigated the ability of human herpesvirus type 2 (HHV-2) to infect primary murine astrocytes *in vitro* and the effect of infection on their mitochondrial network and actin cytoskeleton. In immunofluorescence assays, antibodies against HHV-2 antigens and glial fibrillary acidic protein (GFAP) were used to confirm that the infected cells are indeed astrocytes. Real-time PCR analysis showed a high level of HHV-2 replication in astrocytes, particularly at 168 h postinfection, confirming that a productive infection had occurred. Analysis of mitochondrial morphology showed that, starting from the first stage of infection, HHV-2 caused fragmentation of the mitochondrial network and formation of punctate and tubular structures that colocalized with virus particles. Furthermore, during the late stages of infection, the infection affected the actin cytoskeleton and induced formation of actin-based cellular projections, which were probably associated with enhanced intracellular spread of the virus. These results suggest that the observed changes in the mitochondrial network and actin cytoskeleton in productively infected astrocytes are required for effective replication and viral spread in a primary culture of astrocytes. Moreover, we speculate that, in response to injury such as HHV-2 infection, murine astrocytes cultured *in vitro* undergo transformation, defined *in vivo* as reactive astrocytosis.

## Introduction

Astrocytes are the most abundant cells found in the central nervous system (CNS). They are responsible for regulating brain functions, including neurogenesis and synaptogenesis, as well as controlling blood brain barrier (BBB) permeability and maintaining homeostasis [20]. Several neurotropic viruses (e.g., herpesviruses, enteroviruses, paramyxoviruses, and retroviruses) have evolved a broad spectrum of mechanisms to overcome the BBB and enter the CNS to cause inflammatory diseases such as viral encephalitis. In response to infections, astrocytes generate innate immune responses, which occur through the expression of pathogen-associated molecular patterns (PAMPs) and many immune mediators, including cytokines, chemokines, and type I interferons (IFNs) [2, 12, 14].

Human herpesvirus 2 (HHV-2; herpes simplex virus type 2, HSV-2), a member of the subfamily *Alphaherpesvirinae*, is a ubiquitous human pathogen that is able to cause genital lesions or meningitis. HHV-2 is a neurotropic virus that establishes latent infection in neurons, which can thus serve as reservoirs during subsequent events of virus reactivation. After primary infection of cells of the epithelial lineage, HHV-2 can gain access to sensory neurons, spread to other neurons, and finally enter the CNS. The clinical manifestations of HHV-2 infection in the CNS are classified into several types: meningitis, encephalitis, sacral radiculitis, and myelitis [18, 22]. Although neurons are considered the main target CNS cells for neurotropic herpesviruses, they are also capable of infecting other types of brain cells, including microglia and astrocytes.

It has been reported that astrocytes cultured *in vitro* are susceptible to herpesviruses infection. Productive infection of primary human astrocytes was described previously for human herpesvirus 1 (HHV-1) [1], human herpesvirus 4 (HHV-4; Epstein-Barr virus, EBV) [16], human herpesvirus 5 (HHV-5; human cytomegalovirus, CMV) [13], and human herpesvirus 6 (HHV-6) [7, 10]. As a consequence of infection, astrocytes undergo severe transformation, and a cytopathic effect can be observed that is manifested by a change in cell morphology from a stellate to a globoid shape with a loss of astrocytic projections. Moreover, induction of apoptosis and a progressive decrease in the number of viable cells has been observed in primary human astrocyte cultures infected with HHV-1 [24]. Likewise, HHV-6 infection of human fetal astrocytes results in the activation of apoptosis via the mitochondrial intrinsic pathway [7], and impaired mitochondrial dynamics have also been observed in HHV-1 and HHV-2 infections [5, 6, 24].

Since astrocytes are supportive glial cell components in neural tissue that play a critical role in host defense during viral infection, any alteration in the function of astrocytes might contribute to pathological changes in the CNS and neurological complications. It is known that, in response to CNS damage such as trauma, neurodegenerative diseases, stroke, or viral infection, astrocytes undergo dramatic transformation, referred to as "reactive astrocytosis", which results in progressive alterations in molecular expression, progressive cellular hypertrophy, proliferation, and finally scar formation. Reactive astrocytes can cause disfunction of normal astrocytes and affect their response to inflammation [2].

The aim of our study was to examine the events that occur during HHV-2 infection of primary murine astrocytes *in vitro*. We investigated changes in the actin cytoskeleton and mitochondrial morphology and observed that host cell components, including the actin cytoskeleton and mitochondria, are required for productive replication of HHV-2 in primary murine astrocytes. We have also observed that astrocytes cultured *in vitro* undergo transformation, defined *in vivo* as reactive astrocytosis, as a result of HHV-2 infection.

## Materials and methods

### Primary murine astrocyte culture

**Fig. 1:**
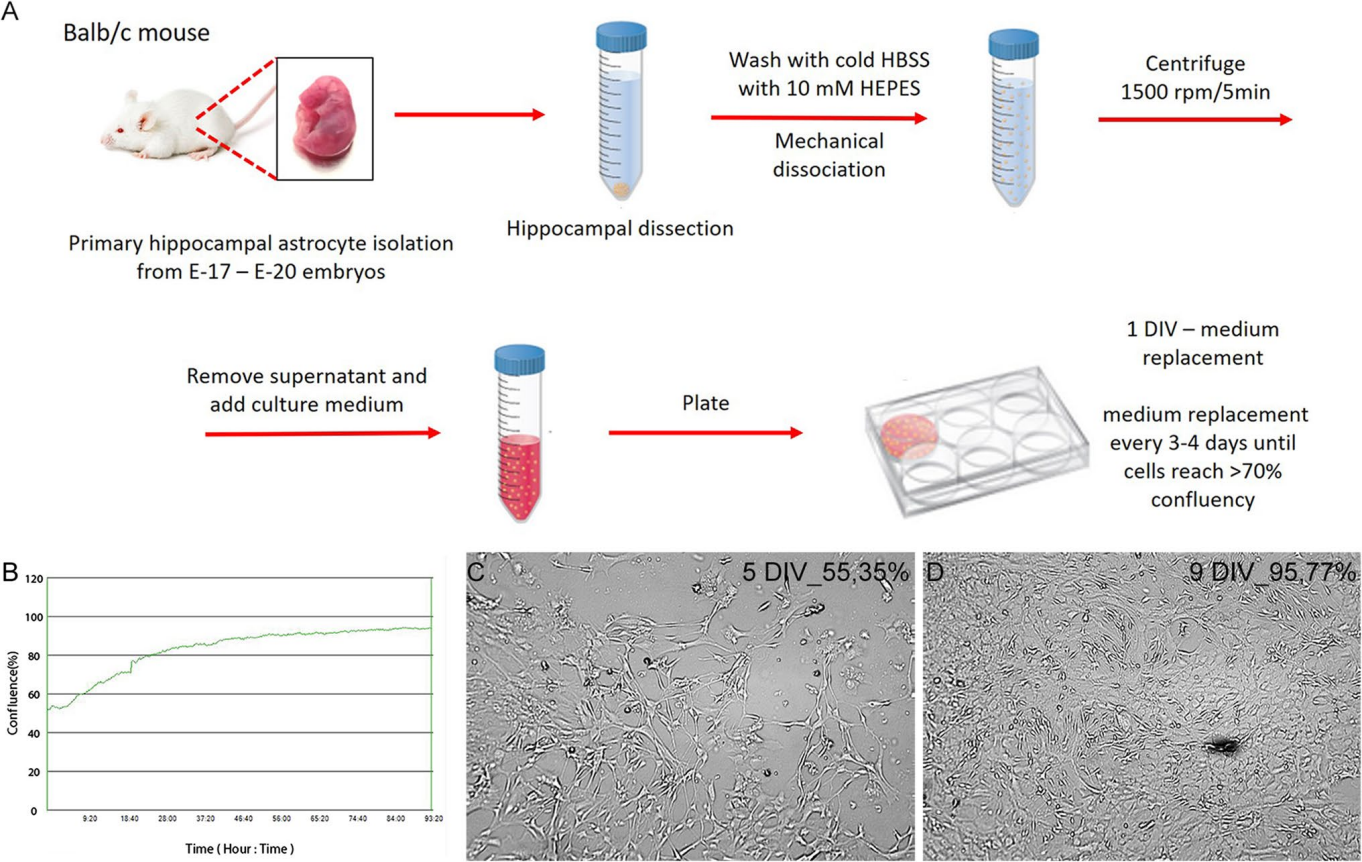
Primary astrocyte culture derived from BALB/c (H-2d) mice. (A) Morphology of mock-infected primary murine astrocytes. (B) Real-time cell growth analysis performed using a JuLI™ Br Live Cell Analyzer. Cultures were observed for 94 hours after initial seeding. Images were recorded every 15 min and analyzed for monolayer confluence. Objective magnification, ×40

BALB/c (H-2^d^) mice, which are genetically susceptible to herpesviral infections, were used to establish a primary culture of murine astrocytes. Briefly, cerebral hemispheres were washed three times in dissection medium (Hanks' balanced salt solution [HBSS] supplemented with 10 mM HEPES [Sigma-Aldrich]) and then transferred to pre-warmed glial growth medium consisting of minimum essential medium containing Earle’s salts with l-glutamine (Gibco, Life Technologies), supplemented with 0.6% glucose, 1% antibiotics (penicillin and streptomycin), and 10% heat-inactivated horse serum (Sigma-Aldrich). Gentle mechanical dissociation was performed using a fire-polished Pasteur pipette. The cell suspension was centrifuged at 1500 rpm for 5 min, and the cell pellet was then resuspended in fresh glial growth medium and seeded onto culture slides coated with poly-D-lysine and laminin at a density of 10^5^ cells per well. After 1 day, the medium was replaced with fresh glial growth medium, which was changed completely every 3–4 days until the cells reached >70% confluency. The purity of the astrocyte cultures was confirmed by glial fibrillary acidic protein (GFAP) labelling. The astrocyte cultures were comprised of cells that were >99% GFAP-positive (Fig. 1A).

### Cell viability assay

Real-time cell growth analysis (cellular growth, morphology, cell density, cell viability) was performed using a JuLI™Br Live Cell Analyser (NanoEnTek), a microscope system for bright-field analysis that enables recording of time-lapse images. Astrocyte growth was observed for 10 days *in vitro* (DIV) with 15-min intervals, and monolayer confluence was analysed according to the manufacturer’s recommendations. The astrocytes were > 90% confluent in all cell cultures (Fig. 1B–D).

### Virus preparation and titration

HHV-2 strain 333 was propagated in confluent Vero cells (ATCC, no. CRL1587) grown in DMEM (Gibco). Cells were infected with HHV-2 at a low multiplicity of infection (MOI) of 0.001 plaque-forming units (PFU)/cell at 37 °C in a humidified 5% CO_2_ atmosphere. At one hour postinfection (h p.i.), the inoculum was removed by aspiration and fresh culture medium was added. HHV-2-infected cells were harvested when 80%–100% of the cells exhibited a cytopathic effect (CPE), and after three cycles of freezing and thawing, cell debris was removed by low-speed centrifugation (800×*g* for 10 min). After purification, virus stocks were stored in small volumes at 80 °C until used [6]. The virus used in this study had a titer of 4 × 10^8^ PFU/ml, as measured by plaque assay in Vero cells.

### Infection of cultured murine astrocytes with HHV-2

In most experiments, primary murine astrocytes (10^6^ cells per well) were grown on microscope coverslips placed in 6-well plates and infected with HHV-2 at 10^8^ PFU/ml (MOI = 0.1) [6]. For real-time PCR analysis, cells were seeded in poly-L-lysine-coated 12-well plates (5 × 10^4^ cells per well). After a 60-min incubation at 37°C in a humidified 5% CO_2_ atmosphere, fresh glial growth medium was added, and the astrocytes were further incubated for 24, 48, or 168 hours at 37°C with 5% CO_2_. Control cells were processed under the same conditions, but they were not infected with HHV-2.

### Quantitative real-time PCR assay (qPCR)

At 1, 24, 48, and 168 h p.i., viral DNA was isolated using a High Pure Viral Nucleic Acid Kit (Roche Diagnostics, Mannheim, Germany) as instructed by the manufacturer. For determining the number of virus copies per reaction, a standard curve was prepared as described by Namvar et al. [11]. The quantity of HHV-2 DNA in all samples was estimated using real-time PCR with TaqMan Universal Master Mix II (Applied Biosystems) according to a previously described protocol [5, 6, 18]. Briefly, for amplification of viral DNA, primers specific for a fragment of the glycoprotein B (gB) gene sequence of HHV-2 were used: HSVgBext_L (GTGATGTTGAGGTCGATGAAGGT) and HSVgBext_R (ACAACGCGACGCACATCAAGGT). The amplicons were then cloned into pGEM-T Easy Vector. The real-time PCR analysis was performed in 96-well plates in a 7500 Real-Time PCR System thermocycler (Applied Biosystems).

### Immunofluorescence assay

To visualize the morphology and distribution of the mitochondrial network, cells were incubated with 100 nM MitoRed (Sigma-Aldrich, St. Louis, MO, USA) for 20 min at 37 °C in a humidified atmosphere of 5% CO_2_ in air. To detect HHV-2 antigen and glial fibrillary acidic protein (GFAP), cells were fixed with 4% paraformaldehyde for 20 min and permeabilized with 0.5% Triton X-100 in phosphate-buffered saline (PBS, Sigma-Aldrich) for 5 min. Before staining, fixed cells were blocked with PBS containing 1% bovine serum albumin (BSA, Sigma-Aldrich) for 40 min to prevent nonspecific binding. The presence of viral antigen was detected using polyclonal rabbit anti-herpes-simplex-virus antibody (Dako, dilution 1:100, 1 h, 37 °C) and Alexa Fluor 488 goat anti-rabbit IgG antibody (Invitrogen, dilution 1:250, 1 h, RT). GFAP was stained with anti-GFAP (Calbiochem, dilution 1:200, 60 min, 37 °C) and Texas Red-X goat anti-mouse IgG (Invitrogen, dilution 1:1000, 60 min, RT) as the secondary antibody. F-actin was labeled with TRITC-phalloidin conjugate (500 ng/ml; Sigma Chemicals) for 60 min. Microtubules were labeled with anti-β-tubulin monoclonal IgG (Sigma-Aldrich, dilution 1:250, 60 min, 37 °C) and detected with Texas Red-X goat anti-mouse IgG (Invitrogen, dilution 1:1000, 60 min, RT). Nuclear DNA was stained with Bisbenzimidine/Hoechst 33258 (Sigma Aldrich, 2 μg/ml) according to the manufacturer’s recommendations. Finally, coverslips were mounted on microscope slides using ProLong Gold Antifade Reagent (Invitrogen).

### Confocal microscopy analysis

Confocal images were acquired using a FluoView FV10i laser scanning confocal microscope (Olympus Poland Sp. z o.o.) equipped with ultraviolet/visible light LD lasers with excitation at 405 nm, 499 nm, 552 nm, and 578 nm to excite Hoechst, Alexa Fluor 488, TRITC, and MitoRed, respectively. Images were captured at 60x magnification and converted to 24-bit tiff files for visualization. Microscopic analysis was performed using FV10i software (Olympus), ImageJ (NIH Image, version 1.53a, USA), and Adobe Photoshop CS6 software (Adobe Systems Incorporated).

### Analysis of mitochondrial network morphology

To analyze the morphology of the mitochondrial network, images obtained from confocal microscopy were analysed using MiNa Single Image macro. This tool enables pre-processing of confocal images to improve their quality and obtain a morphological skeleton for calculating parameters to quantitatively capture the morphology of the mitochondrial network. The number of individual mitochondria, number of networks, mean length of branches/rods, mean number of branches per network, and mitochondrial footprint were evaluated according to the protocol of Valente et al. [23].

### Statistical evaluation

Statistical analysis was performed using GraphPad InStat^TM^ version 3 software (GraphPad Software Inc., San Diego, CA, USA). Data were evaluated by one-way analysis of variance (ANOVA), using the Student–Newman–Keuls multiple comparisons test and the Tukey–Kramer multiple comparisons test. Statistical differences were interpreted as significant at *p* < 0.05 (*), highly significant at *p* < 0.01 (**), extremely significant at *p* < 0.001 (***), and not significant at *p* > 0.05 (ND, not detected). Quantitative data are presented as the mean ± standard deviation (SD) from at least three independent experiments.

## Results

### Permissiveness of primary murine astrocytes to HHV-2 infection

**Fig. 2:**
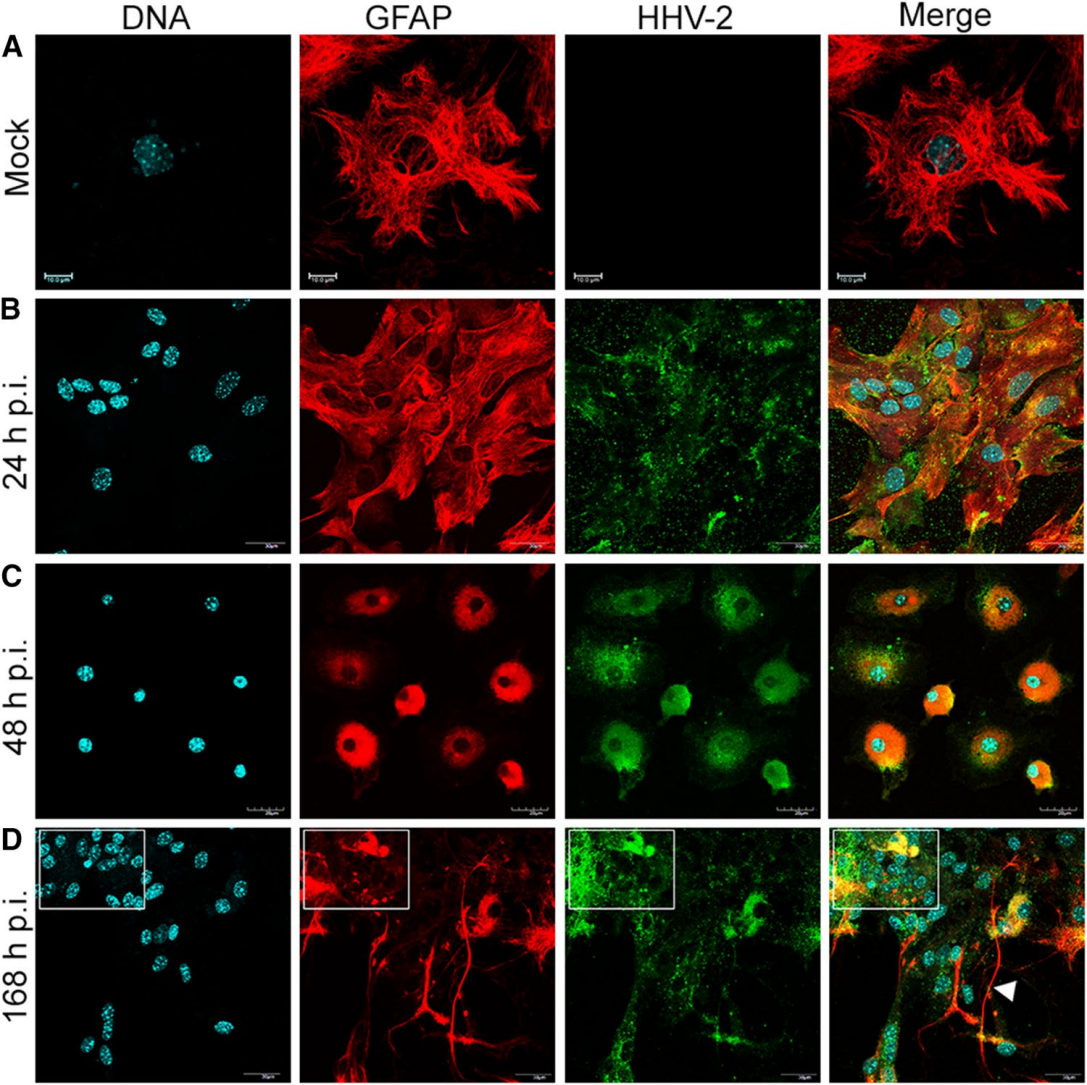
Immunofluorescence images of primary murine astrocytes infected with HHV-2 at 24 (B), 48 (C), and 168 (D) h p.i. Red fluorescence corresponds to GFAP, green to HHV-2 antigens, and blue to DNA. GFAP-positive astrocytes exhibited a radial shape characterized by small soma, long processes, and numerous branches (A). CPE was manifested by changes in cell morphology. Infected astrocytes have a stellate to a globoid morphology with a loss of astrocytic processes at 48 h p.i. (C). The square in panel D indicates a syncytium with multiple nuclei at 168 h p.i. The arrowhead in panel D shows long GFAP-containing projections.

To confirm infection of primary murine astrocytes by HHV-2, a dual immunofluorescence assay for specific viral antigens and glial fibrillary acid protein (GFAP) was performed. Cultured astrocytes were infected with HHV-2 and examined by confocal microscopy at 24, 48, and 168 h postinfection. GFAP-positive astrocytes exhibited a radial shape characterized by small soma, long processes, and numerous branches (Fig. 2A). At 24 h p.i., no significant changes in the distribution of GFAP were observed, while HHV-2 antigen was detected in the whole cytoplasmic compartment (Fig. 2B). At 48 h p.i., we observed that infection of cultured astrocytes with HHV-2 induced a cytopathic effect (CPE), which was manifested by changes in cell morphology. The morphology of the infected astrocytes changed from stellate to globoid, with a loss of astrocytic processes (Fig. 2C). Furthermore, another type of cytopathic effect was observed at 168 h p.i. HHV-2 infection of astrocytes induced cell fusion and formation of multinuclear syncytia (Fig. 2D, boxed region). Most of the viral antigen appeared to be localized in the syncytia, where an intense fluorescence signal was detected. Moreover, individually stained cells also appeared throughout the culture, suggesting that not all infected cells were involved in syncytium formation. However, GFAP staining was much weaker in syncytia than in those individually stained cells.

**Fig. 3:**
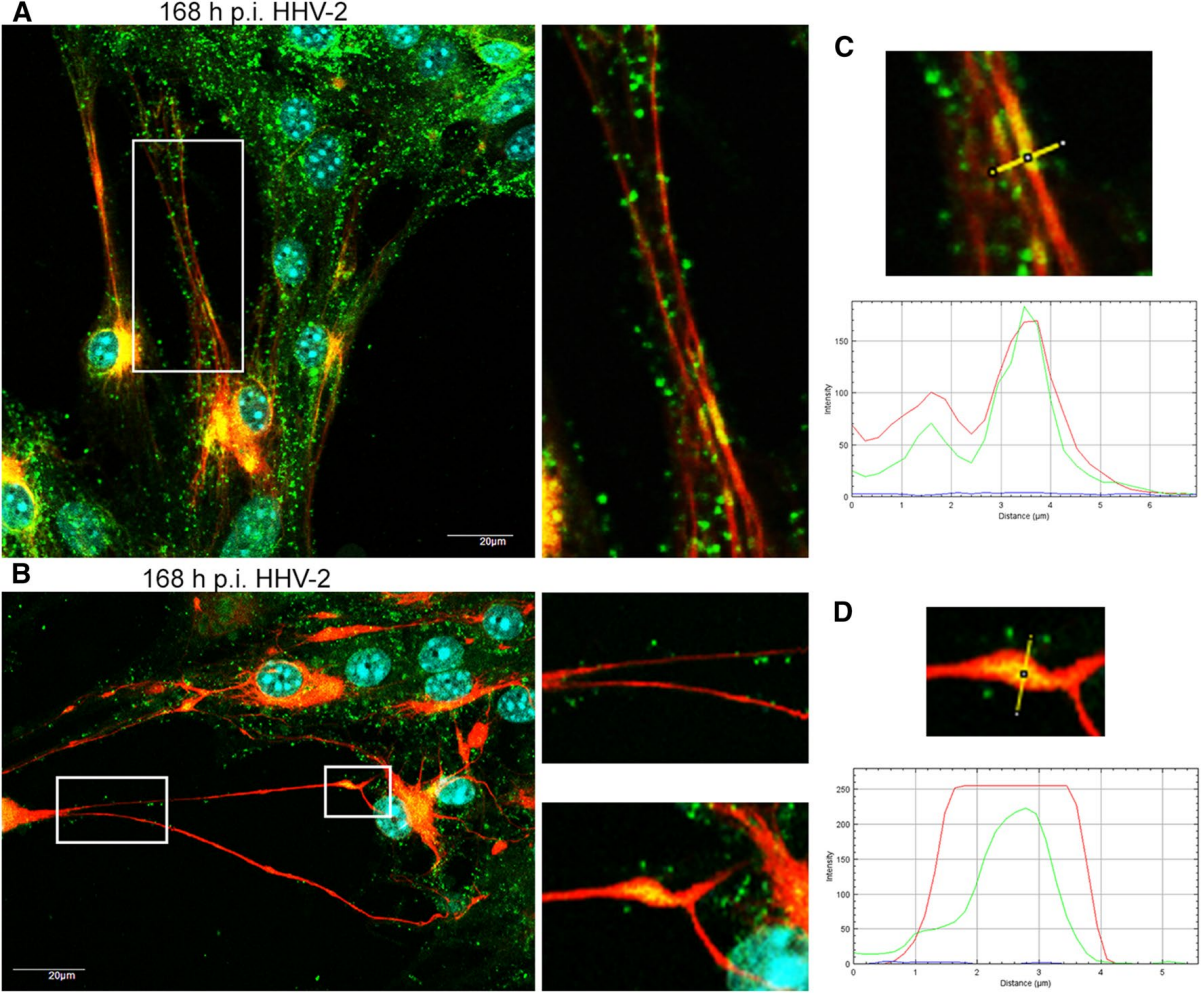
Immunofluorescence images of HHV-2-infected astrocytes, showing viral antigen inside the long GFAP-containing projections (A, B). The magnified images are of the boxed regions. The fluorescence intensity of GFAP (red line) and viral antigen (green line) was measured along the yellow lines (C, D). Red, GFAP; green, HHV-2 antigens; blue, DNA

We also observed that HHV-2 infection induced the formation of long, GFAP-containing projections that established intimate contact with adjacent cells, and virus particles migrated within these protrusions (Fig. 2D, arrowhead; 3A and B). Colocalization of viral antigen with these GFAP-containing projections was confirmed by the linear fluorescence colocalization test, in which the signals corresponding to GFAP and HHV-2-antigen overlapped (Fig. 3C, D). The findings presented here suggest that condensation of GFAP-containing intermediate filaments had occurred. Since expression of GFAP has become a prototypical marker for immunohistochemical identification of astrocytes, we can conclude that the astrocyte cultures, including the infected cells, expressed GFAP, indicating that the infected cells are indeed astrocytes.

### Kinetics of HHV-2 replication in primary murine astrocytes

**Fig. 4:**
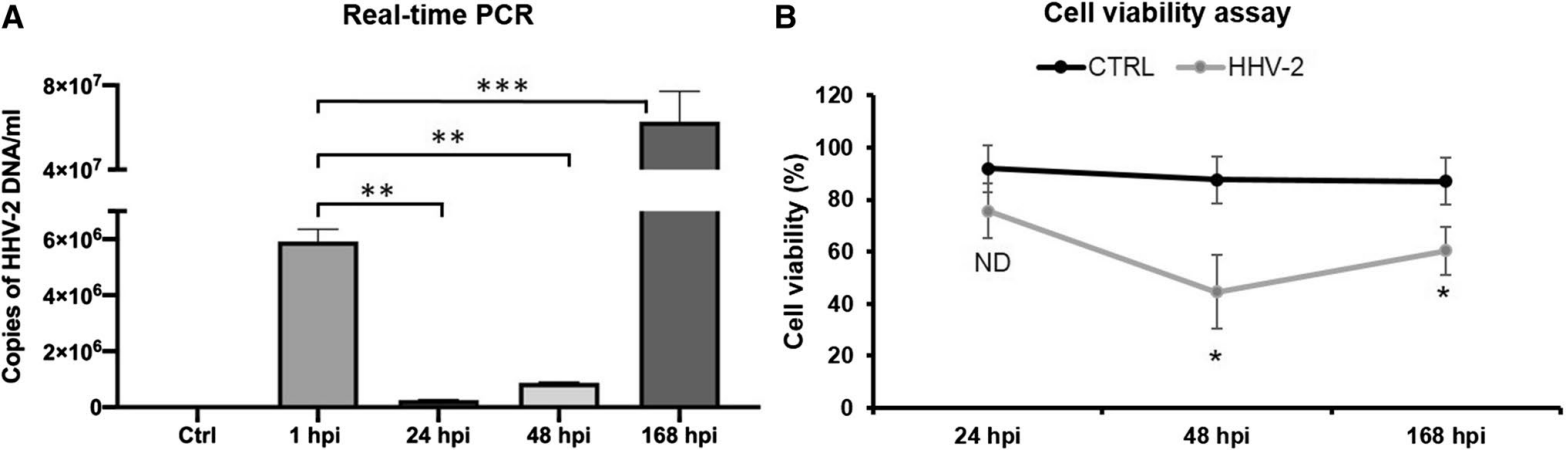
(A) Real-time PCR determination of the viral DNA copy number in primary murine astrocytes during HHV-2 infection. (B) Cell viability assay performed using a JuLI™Br Live Cell Analyser. Results are presented as the mean ± SD of at least three experiments. Statistical comparisons were made between mock-infected cells and HHV-2-infected astrocytes at 24, 48, and 168 h p.i. Statistical differences were interpreted as significant at p < 0.05 (*), highly significant at p < 0.01 (**), extremely significant at p < 0.001 (***) and not significant at p > 0.05 (ND, not detected).

To confirm the infection of the murine astrocyte cultures by HHV-2, viral DNA was extracted at 1, 24, 48, and 168 h postinfection from infected astrocytes and analyzed for HHV-2 sequences by real-time qPCR. Quantitative analysis (real-time qPCR) showed a high level of HHV-2 DNA in primary murine astrocytes: 5.92 ± 0.4 × 10^6^ copies/ml at 1 h p.i. (after adsorption). At 24 (2.44 ± 0.15 × 10^5^ copies/ml; *p* < 0.01,**) and 48 (8.70 ± 0.24 × 10^5^ copies/ml; *p* < 0.01,**) h p.i., a significant decrease in the level of viral DNA was detected in comparison with the results obtained after one hour of adsorption. However, the highest level of viral DNA in astrocytes was detected at 168 h p.i. (6.28 ± 1.31 × 10^7^ copies/ml, *p* <0.001, ***) (Fig. 4A). These results indicate that, despite the initial decrease in the replication level at 24 and 48 h p.i., when compared to 1 h p.i., HHV-2 intensively replicated in the infected astrocytes, as evidenced by the large increase in the viral DNA level at 168 h p.i.

In support of the above observations, we decided to evaluate the viability of the astrocytes after HHV-2 infection, and a cell viability assay was performed with trypan blue using a JuLI™Br Live Cell Analyser. Viability of uninfected astrocytes was maintained at 86-91%. After HHV-2 infection, we observed a significant decrease in cell viability at 48 h p.i. (44.5% ± 14.2 %; *p* < 0.05,*) in comparison with uninfected control cells (88.8 ± 2.7%) (Fig. 2B). It is worth noting that, at that time, the cytopathic effect described above (shrunken and rounded appearance of cells) was also observed. A similar effect was observed at 168 h p.i., and the viability of the cells dropped to 60.3% ± 9.1% (*p* < 0.05,*) (Fig. 4B). This shows that HHV-2 reduces the viability of primary murine astrocytes but does not lead to the death of all cells in the culture, since they apparently are essential for virus replication. These results are consistent with those obtained by confocal image analysis and quantitative PCR and indicate that a productive infection of murine astrocytes had occurred.

### Rearrangement of the actin cytoskeleton during HHV-2 replication

**Fig. 5:**
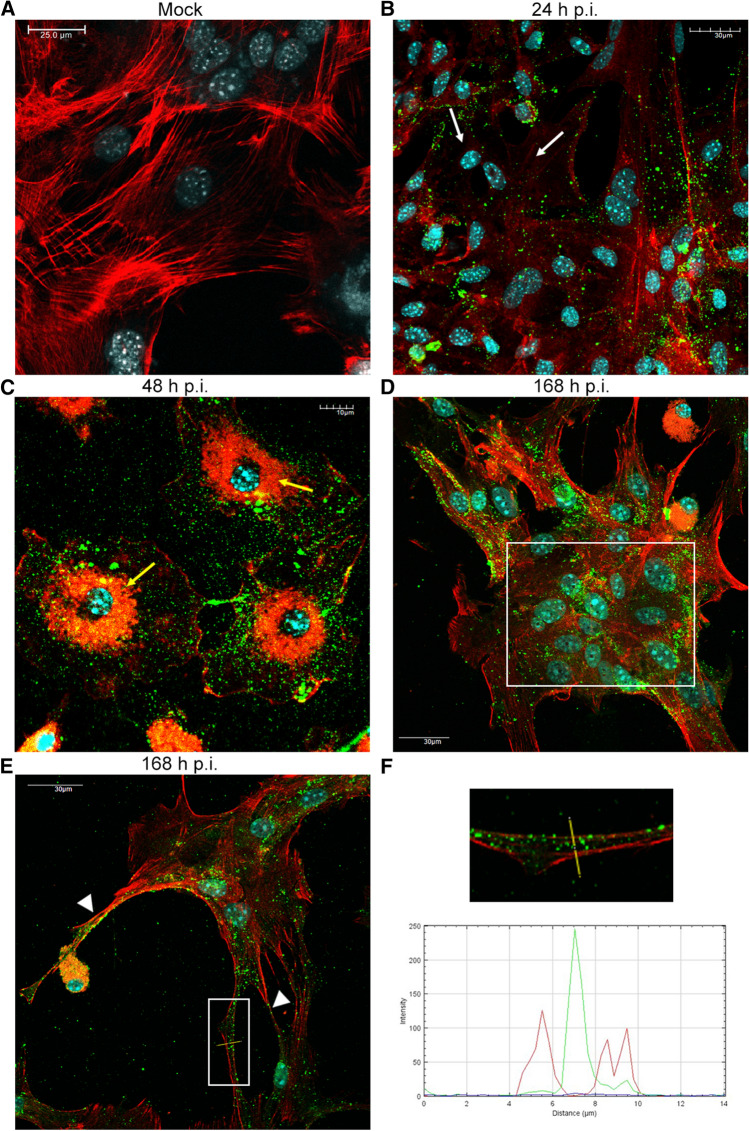
Immunofluorescence images of actin filaments in HHV-2-infected primary murine astrocytes. White arrows in panel B show disruption of microfilaments system within the cytoplasm. Yellow arrows in panel C indicate F-actin condensation in the perinuclear area. The square in panel D indicates a syncytium with multiple nuclei. Arrowheads in panel E indicate actin-rich projections. The square in panel E indicates a region where the fluorescence intensity measurement was performed, as shown graphically in panel F.

The actin cytoskeleton is a highly dynamic structure that plays an essential role in vital cellular processes, such as cell division, migration, and intracellular transport. It is also involved in determining cell size and shape. Since HHV-2 infection led to changes in astrocyte morphology, we investigated the role of actin filaments during HHV-2 infection. The organization of actin filaments in mock- and HHV-2-infected astrocytes was examined by confocal microscopy at 24, 48, and 168 h p.i. In mock-infected astrocytes, actin filaments formed a network of fibers within the cytoplasm. However, the densest distribution was detected in the cell cortex, which is adjacent to the plasma membrane (Fig. 5A). As visualized by confocal microscopy, actin cytoskeleton remodeling was observed in HHV-2-infected astrocytes. At 24 h p.i., HHV-2 caused disruption of the microfilament system and general depolymerization of actin, especially of fibers inside the cytoplasm (Fig. 5B, white arrow). At 48 h p.i., as with GFAP staining, changes in cell morphology were observed—astrocytes were rounded and devoid of astrocytic projections. The actin cytoskeleton underwent drastic reconstruction simultaneously. Disappearance of actin stress fibers in the cortical cytoplasm and F-actin condensation in the perinuclear area were observed (Fig. 5C, yellow arrow). At 168 h p.i., a loss of actin structures inside syncytia, which resulted in a decrease in overall actin staining, was also observed (Fig. 5D, boxed region). In addition to actin stress fiber disassembly, HHV-2 infection promoted the formation of long actin-containing projections from the surface of sparsely plated cells (Fig. 5E**,** arrowheads). Moreover, accumulation of viral antigen inside these projections was detected, which was confirmed by the linear fluorescence colocalization test (Fig. 5F). The above-mentioned structures may contribute to the transmission of HHV-2 virions to adjacent uninfected astrocytes and thus promote virus spread in the cell culture without being exposed to the extracellular environment.

### HHV-2 infection causes disruption of the mitochondrial network and its distribution in astrocytes

**Fig. 6:**
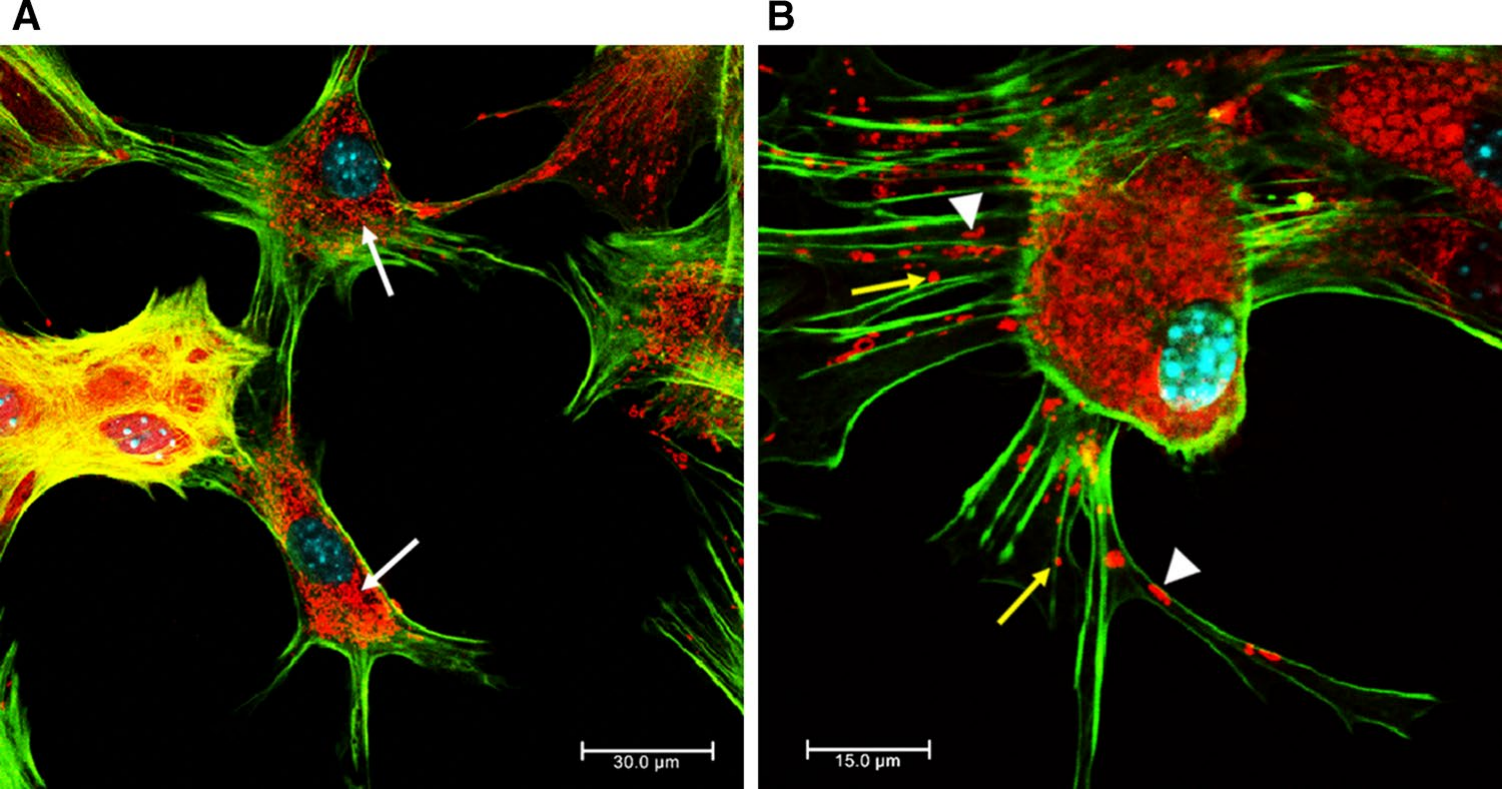
Immunofluorescence images of the mitochondrial network (red fluorescence) and actin filaments (green fluorescence) in mock-infected astrocytes. In mock-infected astrocytes, mitochondria had a tubular morphology and formed a branched and interconnecting network, mainly in the subcellular region (A, white arrows). Yellow arrows indicate single punctate mitochondria, and arrowheads indicate tubular mitochondria localized inside the astrocytic projections (B).

Mitochondria are highly dynamic organelles that are essential for energy production and cell survival. In mock-infected astrocytes, mitochondria had tubular morphology and formed a branched and interconnecting network, mainly in the subcellular region (Fig. 6A, white arrows). Single punctate and tubular mitochondria were also localized inside the astrocytic projections (Fig. 6B).

**Fig. 7:**
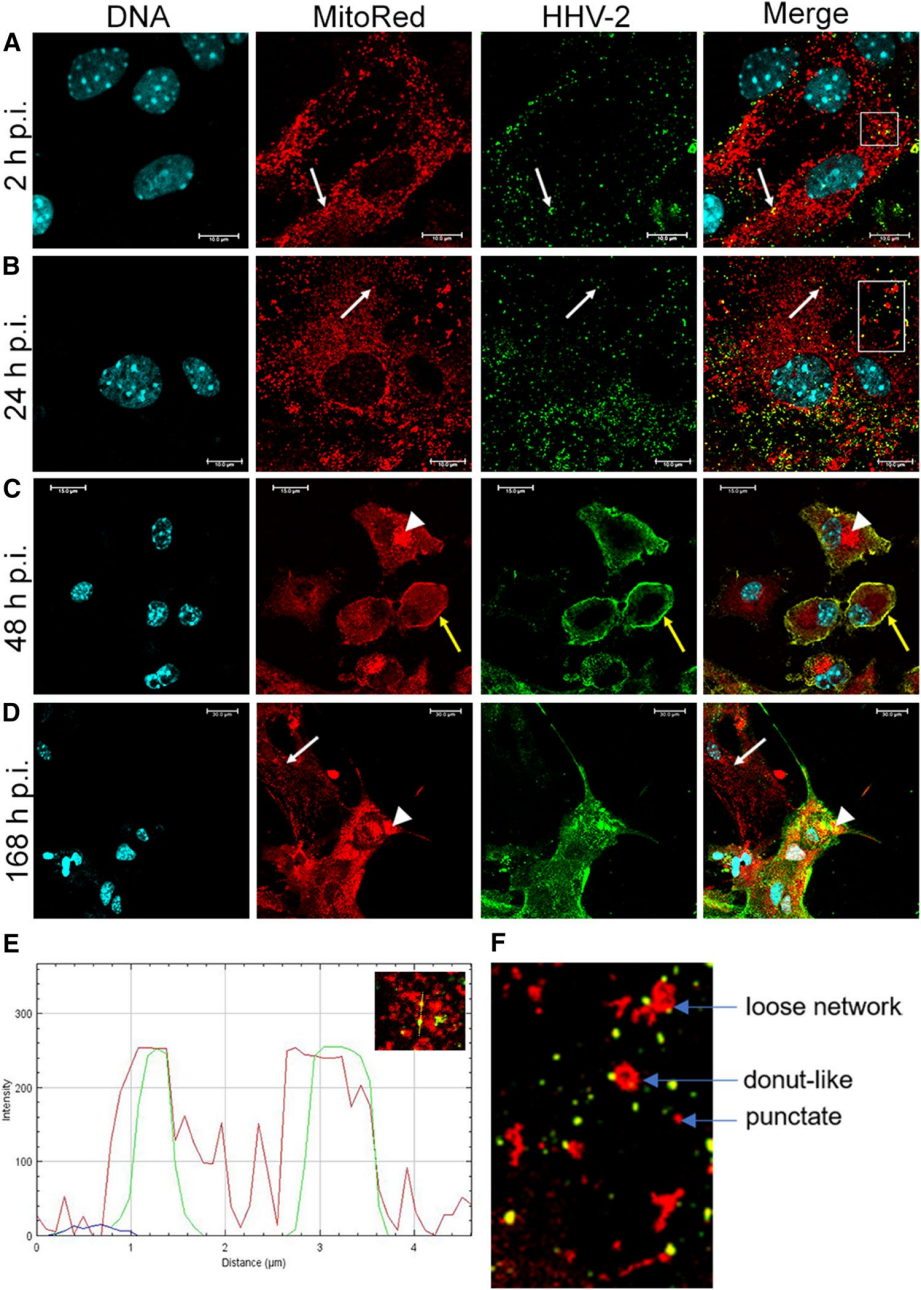
Immunofluorescence images of mitochondrial network morphology in HHV-2-infected astrocytes at 2 (A), 24 (B), 48 (C) and 168 (D) h p.i. Mitochondria, red; HHV-2 antigens, green; DNA, blue. White arrows in panels A and B indicate fragmented, punctate mitochondria colocalized with viral antigens. Yellow arrows indicate viral antigens located at the periphery of the cell (C). Arrowheads in panels C and D indicate mitochondrial aggregates located within the cytoplasm of infected cells. The square in panel A indicates the region where the fluorescence intensity measurement was performed, as shown graphically in panel E. The square on panel B indicates a region with dispersed mitochondria that were present in various forms: a loose mitochondrial network, a donut-like mitochondrion, and a single, small punctate mitochondrion (F).

The influence of HHV-2 infection on the mitochondrial network was monitored starting from 2 h p.i. The interaction between the virus and mitochondria was already observed at an early stage of infection. At 2 h p.i., mitochondria were not branched but had a regular distribution within the cytoplasm. At that time, viral antigen was mainly located at the periphery of the astrocytes, but also within the cytoplasm (Fig. 7A**)**. Moreover, its co-location with mitochondria, confirmed by the linear fluorescence colocalization test, was detected (Fig. 7A, E). At 24 h p.i., the branched network of mitochondria was dispersed within the cytoplasm, and mitochondria were fragmented and disorganized with a loss of connection between them. They also displayed various forms: (i) a loose mitochondrial network, (ii) donut-like mitochondria, and (iii) single, small punctate mitochondria (Fig. 7B, F). Within the cytoplasm, the presence of progeny virions that colocalized with the mitochondrial structures was observed (Fig. 7B, white arrows). At 48 h p.i., due to the change in astrocyte morphology, mitochondria accumulated in the form of aggregates in close proximity to the perinuclear area (Fig. 7C, arrowheads). Furthermore, the viral antigen was mainly located at the periphery of the cell (Fig. 7C, yellow arrows). At 168 h p.i., in the infected astrocytes that had fused to form syncytia, mitochondrial aggregates were also observed (Fig. 7D, arrowheads). However, as stated above, at this time point there were also single infected cells, and within them the mitochondrial network was fragmented, and single punctate or tubular mitochondria were detected. Colocalization of viral antigen with mitochondria was also observed (Fig. 7D).

**Fig. 8:**
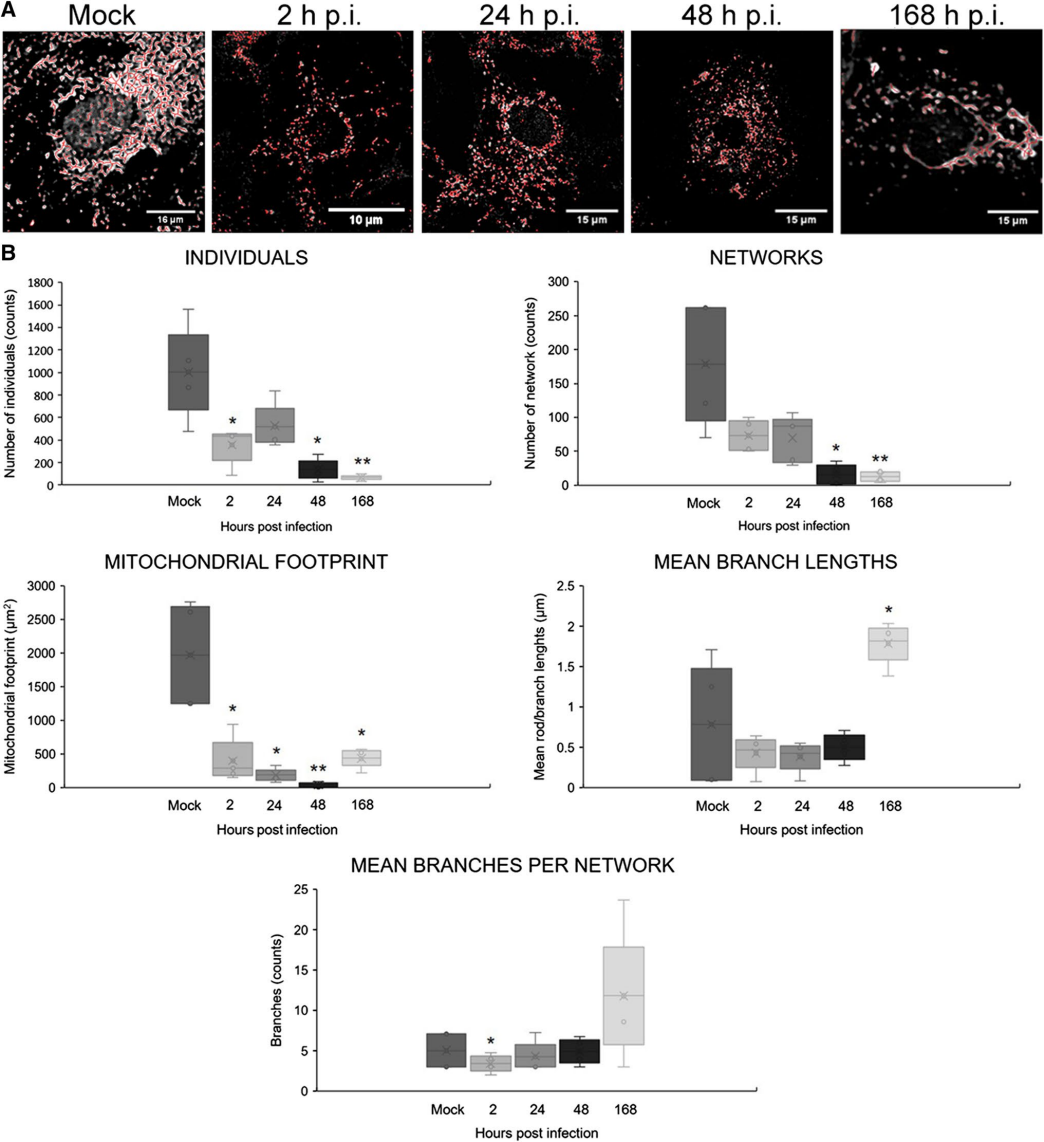
Morphology of the mitochondrial network in HHV-2-infected astrocytes using MiNa Single Image Macro. The number of individual mitochondria, the number of networks, the mean lengths of branches and rod, the mean network size, the mean number of branches per network, and the mitochondrial footprint were measured. (A) Images of single, mock-infected, and HHV-2-infected (2, 24, 48, 168 h p.i.) astrocytes prepared for mitochondrial network feature analysis. The graph shows a summary statistic of mock- and HHV-2-infected astrocytes (each analysis was performed on 10 cells). (B) Box plot showing median values (horizontal lines), first-to-third quartile values (box), and the most extreme values (%). Statistical differences were interpreted as significant at p < 0.05 (*) and highly significant at p < 0.01 (**).

An additional analysis of mitochondrial morphology was performed based on confocal microscopy images using MiNa Single Image macro. Initially, the images were pre-processed to enhance the image quality prior to analysis to provide more accurate results (Fig. 8A). During the infection of the astrocyte cultures with HHV-2, there was a significant reduction in the number of individual mitochondria and mitochondrial networks, as well as in the mitochondrial footprint in comparison to mock-infected cells at all time points tested. The mean length of mitochondrial branches had decreased at 2, 24, and 48 h p.i, but at 168 h p.i., there was an increase in this parameter. Similarly, at 2, 24, and 48 h p.i., the mean number of branches per network was at a level similar to that in mock-infected astrocytes, but after 168 hours of infection, an increase in the number of branches was observed (Fig. 8B). Taken together, our findings indicate that HHV-2 interacts with the mitochondrial network of murine astrocytes during viral infection.

## Discussion

Astrocytes are the most abundant specialized glial cells in the central nervous system (CNS), and they play multiple roles in CNS functions and development. They outnumber neurons by fivefold and have been described as supportive cells that promote neuronal growth and survival. Moreover, in response to a variety of CNS insults, including viral infections, astrocytes are able to express pattern recognition molecules, such as Toll-like receptors, and secrete immune mediators (chemokines and cytokines). Several viruses, including herpesviruses, can infect astrocytes, and serious CNS damage and neurological disorders may occur as a result of infection [2, 14].

Studies investigating herpesvirus infection in astrocytes have mainly been conducted on primary human fetal astrocytes and have therefore required special ethical approval [4, 7, 10, 24]. Animal models are widely used in research to investigate the mechanisms of HHV infection and pathogenesis. In the present study, we used primary astrocytes, but these cells were isolated from BALB/c mice. A similar *in vitro* model was utilized previously for the study of the mechanisms of HHV-1 infection and cell death in primary glial cells [1]. It is worth noting that although mice are not natural hosts for HHV-1 and HHV-2, they are susceptible to infection, and replication of both viruses occurs in the nervous system. For this reason, primary murine astrocytes are a valuable model for investigation of the mechanism of herpesviral infection in astrocytes.

Herpesviruses have the capacity to infect and replicate in astrocytes. Productive infection of astrocytes has been described for alphaherpesviruses (HHV-1) [1], betaherpesviruses (HHV-5 and HHV-6) [7, 10, 13], and gammaherpesviruses (HHV-4) [16]. In order to study the susceptibility of primary murine astrocyte cultures to HHV-2 infection, an immunofluorescence assay for specific viral antigen and glial fibrillary acidic protein was performed. We observed that infected cells were stained with antibodies against the HHV-2 antigen and GFAP, indicating that they were indeed astrocytes. The infected cells showed various cytopathic effects, which were manifested by changes in cell morphology at 48 h p.i. and by the formation of syncytia between infected and adjacent cells at 168 h p.i. A similar cytopathic effect in astrocytes was reported previously for HHV-1 and HHV-6. In primary human astrocytes infected with HHV-1, Wnęk et al. [24] observed a progressive reduction in the number of viable cells as well as changes in the morphology of astrocytes, which changed from stellate to globoid at 48 and 72 h p.i. Infection of primary human fetal astrocytes with HHV-6 has been shown to induce cell fusion and formation of giant syncytia [10]. To confirm infection of the primary murine astrocytes by HHV-2, we analysed viral replication kinetics by qPCR. Initially, after an hour of adsorption, viral DNA levels dropped significantly at 24 and 48 h p.i. This was probably due to the decrease in cell viability that was observed using a JuLI™Br Live Cell Analyser. However, it is worth noting that HHV-2 infection did not lead to the death of all of the cells in the culture, as was indicated by the highly significant increase in viral DNA level observed at 168 h p.i. In astrocyte culture, a proportion of viable cells remained in which the virus could effectively replicate. Based on these results and those obtained from confocal image analysis, we assume that primary murine astrocytes were productively infected by HHV-2.

In order to optimize viral replication and production of new virions, viruses have evolved myriad strategies to alter the normal functions of the infected cell. One of these alterations is the reorganization of the actin cytoskeleton at each stage of the viral life cycle, including entry, assembly, and egress [15, 19]. In the current study, using confocal microscopy, we observed that HHV-2 infection results in the disruption and rearrangement of actin network. In general, the infection caused partial or total disappearance of actin stress fibers within the cytoplasm. Moreover, we observed that HHV-2 induced formation of actin-based cellular projections during the late stages of infection (168 h p.i), and this was probably associated with enhanced intracellular spread of newly assembled virions. Similar extensions were observed using GFAP staining. Apart from its status as a classical marker for astroglia, GFAP also performs many important functions in astrocytes, including the maintenance of their specific morphology, control of cell migration, maintenance of the stability of processes, and participation in astrocyte-neuron interactions and cell-cell communication. It is also the main component of intermediate filaments of the cytoskeleton of astrocytes [21]. In response to injury of the CNS caused by trauma, neurodegenerative disease, or viral infection, GFAP plays crucial role in the development of reactive astrocytosis [2, 21]. During astrocytosis, increased proliferation, morphological changes (such as hypertrophy of the cell soma), increased expression of GFAP, and processes filled with GFAP-containing filaments are observed. In our study, we discovered that HHV-2 induced formation of long GFAP-containing projections that established intimate contact with adjacent cells. Virus particles could potentially migrate within these protrusions. The actin cytoskeleton was also found to be involved in the formation of these projections. It is known that, as a result of viral infection, astrocytes become activated [2]. This was also confirmed in a study by Gumenyuk et al. [8] showing that HHV-1 infection leads to the development of intense reactive astrogliosis in murine brain. Based on these findings, we speculate that, in response to viral infection, astrocytes cultured *in vitro* undergo transformation, defined *in vivo* as reactive astrocytosis, in which the long cell projections containing cytoskeletal elements serve to propel newly produced virions from cell to cell.

Rearrangements of the actin cytoskeleton affect the size and shape of the cells, its functions, and the intracellular distribution of organelles, including mitochondria. Mitochondria are essential for energy production and cell survival. In neural tissue, mitochondria can be transferred from astrocytes to neurons, and this contributes to neuroprotection and neurorecovery after trauma such as stroke [3, 9]. Mitochondria are also used by viruses to obtain the energy necessary for their replication. Murata et al. [17] reported that during HHV-2 infection of epithelial cells, mitochondria cluster in the vicinity of the cell nucleus, where accumulation of the viral tegument proteins pUL41 and pUL46 was also observed. For that reason, in the current study, we investigated the influence of HHV-2 on the mitochondrial network and its distribution in primary murine astrocytes. Our results showed that, starting from the first stage of HHV-2 infection, the interactions occurred between the virus and mitochondria. In addition to fragmentation of the mitochondrial network, we also observed colocalization of viral antigen with mitochondria, confirmed by the linear fluorescence colocalization test. These findings confirmed that the mitochondria were active and that their probable role was to supply ATP for HHV-2 morphogenetic processes. In additional analysis of mitochondrial network morphology, performed using MiNa Macro Tools analysis, we again observed that HHV-2 significantly disrupted the mitochondrial network. The branched network of mitochondria was dispersed with a loss of connections between them, as was evidenced by a significant reduction in the network and detection of various forms of mitochondria, such as a loose mitochondrial network and punctate or tubular mitochondria. Division of the mitochondrial network seems to be important for HHV-2 and may result in transport of mitochondria to cell compartments where a large amount of energy is required. The significant role of mitochondria in HHV-2 infection of primary murine neurons was shown previously by Cymerys et al. [6], who showed that, regardless of cell type, mitochondria were required for effective replication of the virus.

In conclusion, our results indicate that HHV-2 can productively infect and replicate in primary murine astrocytes *in vitro*. During the infection, we observed fragmentation of the mitochondrial network as well as an increase in the number of punctate and tubular mitochondria, which colocalized with viral antigen. These findings support the hypothesis that these mitochondria supply energy that is required for viral replication. Long projections containing cytoskeletal elements—actin filaments and GFAP—contribute to the direct spread of newly formed virions to adjacent cells without them being exposed to the extracellular environment. Moreover, changes observed in HHV-2-infected culture of murine astrocytes indicate that they undergo a transformation known as reactive astrogliosis.

## Data Availability

The data that support the findings of this study are available from the corresponding author upon reasonable request.
